# Simple Wound Irrigation in the Postoperative Treatment for Surgically Drained Spontaneous Soft Tissue Abscesses: Study Protocol for a Prospective, Single-Blinded, Randomized Controlled Trial

**DOI:** 10.2196/resprot.7419

**Published:** 2017-05-01

**Authors:** Annika Rühle, Florian Oehme, Katja Börnert, Lana Fourie, Reto Babst, Björn-Christian Link, Jürg Metzger, Frank JP Beeres

**Affiliations:** ^1^ Lucerne Cantonal Hospital Surgery Department Lucerne Switzerland

**Keywords:** skin and soft-tissue infections, recurrent infection, wound irrigation, surgical draining of abscesses, aftercare

## Abstract

**Background:**

Skin abscesses are a frequent encountered health care problem and lead to a significant source of morbidity. They consequently have an essential impact on the quality of life and work. To date, the type of aftercare for surgically drained abscesses remains under debate. This leads to undesirable practice variations. Many clinical standard protocols include sterile wound dressings twice a day by a home-care service to reduce the chance of a recurrent wound infection. It is unknown, however, whether reinfection rates are comparable to adequate wound irrigation with a nonsterile solution performed by the patient. Our hypothesis is that simple wound irrigation with nonsterile water for postoperative wound care after an abscess is surgically drained is feasible. We assume that in terms of reinfection and reintervention rates unsterile wound irrigation is equal to sterile wound irrigation.

**Objective:**

The primary aim of this study is therefore to investigate if there is a need for sterile wound irrigation after surgically drained spontaneous skin abscesses.

**Methods:**

In a prospective, randomized controlled, single-blinded, single-center trial based on a noninferiority design, we will enroll 128 patients randomized to either the control or the intervention group. The control group will be treated according to our current, standard protocol in which all patients receive a sterile wound irrigation performed by a home-care service twice a day. Patients randomized to the intervention group will be treated with a nonsterile wound irrigation (shower) twice a day. All patients will have a routine clinical control visit after 1, 3, 6, and 12 weeks in the outpatient clinic. Primary outcome is the reinfection and reoperation rate due to insufficient wound healing diagnosed either at the outpatient control visit or during general practitioner visits. Secondary outcome measures include a Short Form Health Survey, Visual Analog Scale, Patient and Observer Scar Assessment Scale, Vancouver Scar Scale, and the EurolQol 5-Dimension Questionnaire. Those questionnaires will be completed at the outpatient control visits.

**Results:**

The trial was started in June 2016 and enrolled 50 patients by article publication. Regarding the adherence to our protocol, we found 10% of loss to follow-up until now. Only 2 patients needed reoperation and only 1 patient needed a change of treatment (antiseptic therapy). Most patients are happy with their randomized treatment but as expected some patients in the sterile group complained about timing problems with their working hours and home-care service appointments. Most patients in the nonsterile group are satisfied being able to take care of their wounds independently although some patients still depend on the home-care service for the wound dressing. We are hoping to have enrolled enough patients by summer 2017. The follow-up will take until autumn 2017, and study results are expected to be published by the end of 2017. This trial is solely supported by the cantonal hospital of Lucerne.

**Conclusions:**

Nonsterile wound irrigation is more likely to be carried out independently by the patient than sterile wound irrigation. Therefore, if nonsterile wound care shows comparable results in terms of reinfection and reintervention rates, patient independence in the aftercare of surgically drained abscesses will increase, patients can return to work earlier, and health care costs can be reduced. In a preliminary, conservative estimation of health care costs, an annual savings of 300,000 CHF will be achieved in our hospital.

**Trial Registration:**

German Clinical Trials Register DRKS00010418; https://drks-neu.uniklinik-freiburg.de/ drks_web/navigate.do?navigationId=trial.HTML&TRIAL_ID=DRKS00010418 (Archived by WebCite at http://www.webcitation.org/6q0AXp5EX)

## Introduction

Skin abscesses are frequent health care problems that often affect young and vital patients. Abscesses can be a significant source of morbidity and can mean an essential limitation of quality of life as well as an incapacity to work [1]. Therefore, timely and sufficient surgical drainage and aftercare is an essential part of care, not only to prevent potential life-threatening complications due to inappropriate therapy (eg, necrotizing fasciitis, toxic shock syndrome, Ludwig’s angina) but also to enable a normal social life and a return to work as soon as possible.

The current literature shows great national and international variability of the surgical method, with incision, excision, or spindle-shaped opening and without essential evidence for the superiority of one of the methods [2,3]. Furthermore some surgeons prefer a primary wound closure with or without antibiotic therapy [4-7].

Regarding the aftercare for open wound treatment, the diversity continues and literature can be found on wound irrigation with sterile fluids, unsterile fluids [8], and antiseptic fluids [9]; antibiotic therapy [5,6]; and use of different wound dressings such as silver-containing hydrofiber [10], hyaluronate hydrogel [11], and essential oils [12]. These different treatment options are combined in various ways, and so far the results regarding reinfection rates and failure of therapy seem to be comparable.

For superficial perianal abscesses, a German consensus grade S3 (evidence- and consensus-based) guideline is available [13] suggesting surgical drainage without specifying the type of drainage (excision, incision, or spindle-shaped opening) due to limited data. For the aftercare, prospective randomized trials are lacking but the consensus recommends periodic wound irrigation with antiseptic fluid or subsequent antibiotic therapy only being necessary in individual cases [14]. Patients are often instructed to change the dressing twice a day and irrigate the wound with a sterile solution. For sterile treatment, the assistance of a home-care service is often needed, which can impair the social life due to frequent appointments. Therefore, the question arises if an alternative method could at least support the independence of the patient and lead to sufficient wound treatment without regular appointments and sterile wound irrigations.

Considering a trend toward cost-effective medicine and a reasonable use of medical infrastructure, it is questionable whether every wound needs to be irrigated with a sterile solution twice a day by a special home-care service. We consider the nonsterile wound irrigation (carried out in the shower) as a potential treatment option that offers independence for the patient. It is expected that patients can carry it out independently or with the help of a family member. Additionally, this could lead to an earlier return to work [8].

We hypothesize that simple wound irrigation with nonsterile water for postoperative wound care is feasible after surgical draining of abscesses. Moreover, we postulate that wound irrigation in the shower (simple wound irrigation) is equal to sterile wound irrigation accomplished by special home-care service teams in terms of reinfection and reintervention rates. Additionally, we claim that patients who irrigate the wound using nonsterile water (shower irrigation) will be more independent and that the overall costs are less compared to sterile water irrigation due to less frequent consultations of the home-care services.

To our knowledge, this is the first prospective randomized trial evaluating the value of postoperative wound irrigation using nonsterile solution in surgically drained abscesses.

## Methods

### Study Design

Based on a noninferiority design, we designed a prospective, randomized, controlled, single-blinded, single-center trial. We present the study protocol in accordance with the Standard Protocol Items: Recommendations for Interventional Trials guidelines [15].

### Study Population

The study includes patients treated for spontaneous soft tissue abscesses in the largest nonuniversity hospital in Switzerland.

Patients with a primary superficial abscess presenting at the emergency department will be eligible for the inclusion if they fulfill all of the followed criteria:

- Soft tissue abscess with an indication for surgical drainage
- 18 years or older
- Sufficient understanding of spoken and written German
- Signed informed consent
- Hospitalization of at least one night to ensure sufficient teaching from the nurse concerning wound management (according to the study protocol)

We will exclude patients if they meet at least one of the following exclusion criteria:

- Patients presenting with residual abscesses, abscesses located on the head, or abscesses with a confirmed fistula
- Patients suffering from immunodeficiency (eg, HIV infection, leukemia)
- Patients taking autoimmune therapy
- Patients not willing or able to sign the informed consent
- Patients with psychiatric conditions
- Patients not able to return for appointments at 1, 3, 6, and 12 weeks

### Preliminary

Essential prestudy preparations are needed to ensure proper inclusion of patients and correct documentation of the preoperative situation.

#### Defining Period

**Figure 1:**
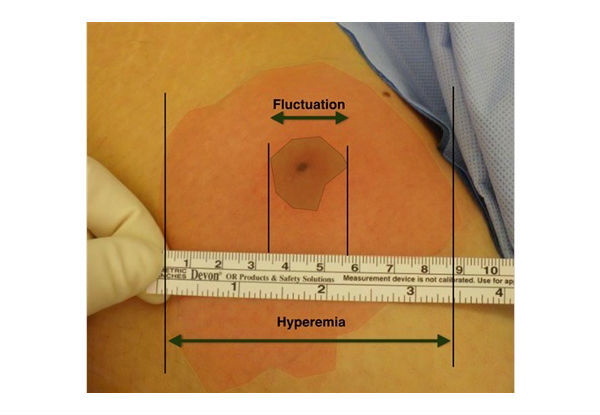
Definition of the area of hyperemia (red) and fluctuation (central frame) with a ruler to indicate the size of the areas.

Standard preoperative documentation of the abscess and its surrounding is necessary to ensure sufficient surgical drainage and to enable comparability between both groups. An abscess was defined as an enclosed collection of liquefied tissue, known as pus, affecting the subcutaneous tissue and representing the immune defense reaction of the body against foreign material or bacteria. As seen in Figure 1, the abscess consists of (1) the area of fluctuation caused by the collection of liquefied tissue, (2) the area of cellulitis caused by hyperemia due to an immune reaction of the body, and (3) the area of induration caused by the hardening of the soft tissue due to the inflammation.

We also defined provisions that needed to be followed to ensure proper surgical drainage during our trial: (1) the incision of the skin has to be at least as long as the fluctuation, (2) the incision of the skin has to be spindle-shaped to ensure a wide opening of the abscess cavity, and (3) the spindle-shaped incision has to include at least two-thirds of the width of the fluctuation.

#### Implementation Period

An educational period teaching basic facts about abscesses will be initiated before the inclusion period starts. All surgeons treating the above mentioned conditions will participate in a special lecture about the pathophysiology, diagnosis, and management of abscesses. We defined the necessity for documentation of the following parameters (see Figure 1): the diameter of the fluctuation and hyperemia and photo documentation with a scale. Finally, everyone involved received handouts containing all relevant information.

### Randomization

Prior to the operation, patients are being randomized either to the control or the intervention group using sealed envelopes. The randomization will be carried out after the signed informed consent is obtained.

#### Control Group (Group I)

Patients belonging to the control group will be treated according to our current protocol, including sterile wound treatment supported by a home-care service. We recommend a standard of sterile wound irrigation twice a day using either Ringer’s lactate or normal saline solution. The wound, including the whole cavity, should be irrigated 3 to 5 times with a 20 mL syringe, treating the whole wound surface with gentle pressure. Thereafter, a moist and unfolded gauze is inserted in the cavity. The unfolded gauze develops a bigger osmotic pressure and therefore ameliorates the suction effect on the cavity. Subsequently the wound should be dressed in a sterile way using gauze and tape to fix the dressing. The home-care service is informed with a standard form.

#### Intervention Group (Group II)

Patients belonging to the intervention group are treated according to a modified aftercare protocol. We recommend shower irrigation for the nonsterile treatment twice a day. The patient is recommended to use a shower with room temperature water and irrigate the entire wound cavity with gentle pressure for 1 to 2 minutes using water only. The use of shower gel, soap, or shampoo is forbidden. After wound irrigation, the dressing of the wound with a moist and unfolded gauze followed by a dry nonsterile gauze and tape is carried out similar to the control group.

The first postoperative wound dressing is changed by the nurse and surgeon on the ward. The patient and/or family members are trained to adequately manage the wound and change the dressings. In case the patient is unable to change the dressing alone, the same home-care service is requested to support the patient. The home-care service is informed with a standard form.

### Outcome

As we present a noninferiority approach, the purpose of this analysis is to show comparable results in the intervention group in terms of reinfection and reoperation rates.

#### Primary Outcome

We defined the primary endpoint as a reinfection or a reoperation after an abscess is surgically drained. A reinfection was defined as a persisting or new reddened wound with signs of induration and fluctuation. If there were any signs of purulent exudation with the above-mentioned signs, we considered the wound to be reinfected, and a reoperation was performed. A reoperation was defined as any operation performed in the same region within the time frame of the study control (12 weeks postoperative).

#### Secondary Outcome

Secondary outcomes are measured using specific scar scores as well as nonspecific outcome tools as shown in Table 1.

##### Specific Scar Tools

The Vancouver Scar Scale (VSS) [16], first described by Sullivan in 1990, assesses 4 variables: vascularity, height/thickness, pliability, and pigmentation. These variables are assessed by the surgeon without the perception of the patient concerning his or her scars.

The Patient and Observer Scar Assessment Scale (POSAS) includes subjective symptoms of pain and pruritus of the scar. It consists of 2 numerical scales: the patient scar assessment scale and the observer scar assessment scale. It assesses vascularity, pigmentation, thickness, relief, pliability, and surface area, and it incorporates patient assessments of pain, itching, color, stiffness, thickness, and relief [17].

##### Nonspecific Tools

Health status measurement will be performed using the Short Form Health Survey (SF-36) in all patients. The SF-36 is a 36-item, patient-reported survey of patient health. The questionnaire includes 8 sections (vitality, physical functioning, bodily pain, general health perceptions, physical role functioning, emotional role functioning, social role functioning, and mental health) and in each a score ranges between 0 and 100. The score is proportional to the outcome, with the best possible result of 100 [18,19].

The EQ-5D is a standardized measure of health status developed by the EuroQol Group. The 5-dimension, 5-level questionnaire consists of 2 pages: the EQ-5D descriptive system and the Visual Analog Scale (VAS). The descriptive system comprises 5 dimensions (mobility, self-care, usual activities, pain/discomfort, and anxiety/depression) and each dimension has 5 levels (no problems, slight problems, moderate problems, severe problems, and extreme problems). Additionally, the VAS records the respondent’s self-rated health on a 20 cm vertical visual analog scale with endpoints labeled “the best health you can imagine” and “the worst health you can imagine.”

A self-designed questionnaire was made to investigate the expenditure during the home-care service visits. Questions concerning the time each home-care service person has spent with wound dressing, type of material (gauze, tape, solution, syringes), and amount of time spent are being recorded. In addition, questions concerning time and frequency of family doctor visits and duration of inability to work will be documented.

A medical questionnaire answered by the surgeon in the outpatient clinic documents the size of the wound cavity. Additionally, the surgeon needs to comment on whether there are signs of cellulitis (defined as flushed tissue with signs of induration), the quality of the wound (cavity and edges), any exudation from the wound or signs of persisting fluctuation (liquid-filled cavities).

### Outpatient Control Visits

All patients receive a standard postoperative outpatient control visit after 1, 3, 6, and 12 weeks. All questionnaires except the VSS and the POSAS will be answered during each visit as shown in Table 1. The VSS and POSAS will be used at the 12-week control visit as definite wound healing is needed to answer these questionnaires. As this is a single-blinded trial, it is essential to ensure blinding of the investigator at the outpatient control visit. All outpatient follow-ups will be carried out by two blinded surgeons investigating the wound and filling out the questionnaire.

**Table 1:** Timeline of outcome measurements.

		Study period					
		Enrollment	Allocation	Outpatient control			
		ED^a^	Operation	Week 1	Week 3	Week 6	Week 12
Enrollment							
	Inclusion criteria	X					
	Informed consent	X					
	Randomization	X					
	Patient baseline characteristics	X					X
Interventions							
	Control group	X	X	X	X	X^b^	X
	Intervention group	X	X	X	X	X^b^	X
Assessments							
	SF-36^c^	X		X	X	X^b^	X
	VAS^d^	X		X	X	X^b^	X
	EQ-5D^e^	X		X	X	X^b^	X
	Medical questionnaire	X		X	X	X^b^	X
	VSS						X
	POSAS						X

^a^Emergency department.

^b^Optional outpatient control, only necessary if wound still open at 3 weeks control.

^c^SF-36: Short Form Health Survey.

^d^VAS: Visual Analog Scale.

^e^EQ-5D: EuroQol 5-Dimension Questionnaire.

^f^VSS: Vancouver Scar Scale.

^g^POSAS: Patient and Observer Scar Assessment Scale.

### Statistical Analysis

Statistical analysis will be performed using SPSS version 21 (IBM Corp). The mean and standard deviation will be calculated and reported for basic patient-related data. Primary analysis will be carried out comparing frequency of reintervention between the intervention and control groups. Moreover, we will try to identify risk factors as basic patient-related data or surgery-associated factors (time of surgery, operation time, experience of the surgeon) using regression models.

Secondary clinical outcome measures will be presented in means with the corresponding standard deviation. The intervention group and the control group will be compared using mean value analysis models (*t* test; multifactorial mean value analysis). We will assume that data missing will be random; therefore, missing data will not be imputed, as we will use the mixed model approach for the longitudinal analyses.

### Sample Size Calculation

Based on international publications, we estimate a 15% reinfection rate as statistically significant [20-22]. The literature remains inconsistent concerning reinfection rates in surgically drained abscesses. Based on these findings and using a power of 0.80 and alpha failure of .05 we performed a sample size calculation. Based on a noninferiority approach, we calculated 58 patients were needed in each treatment arm. We added 10% loss of follow-up to our power calculation (58×1.10=64 patients in each treatment arm) and determined 128 patients need to be included in our study.

### Calculation of Cost Savings

Since nonsterile wound care is easier to perform, we presume that patients in this group are more likely to perform their wound care without the help of a home-care service. Since the dressing in some cases cannot be performed independently and some patients may not have any family member to help, some patients in the nonsterile group may need assistance by a home-care service as well. Also, some patients or family members might feel the need for supervision in the beginning of the wound care, which will be carried out by the home-care service. We assume that on average 1 week of assistance will be enough time and therefore calculated 1 week of home-care service for the nonsterile group.

In contrast, we think that patients in the sterile group are not likely to be able to perform the wound care on their own. Also family members might not be able to provide adequate assistance in sterile wound irrigation and therefore patients are more likely to depend on the home-care service for the entire open wound treatment period. We assume that an average treatment will last 4 weeks.

The estimated time for a proper wound treatment by the home-care service is 20 minutes. Wound treatment is carried out twice per day, totaling 280 minutes each week. The home-care service in the city of Lucerne costs 65 CHF (Swiss Francs) per hour, leading to 305 CHF per week.

If an average treatment lasts 4 weeks and patients from the nonsterile group are thought to be independent after 1 week while patients from the sterile group will need assistance for 4 weeks, a savings of 915 CHF for each patient in the nonsterile group can be assumed solely based on the health care costs. Over the last year, 320 patients with abscesses have been treated at our hospital. Changing our postoperative treatment strategy from a sterile to nonsterile aftercare would save approximately 300,000 CHF annually solely on medical expenses.

### Ethical Considerations

The study design is in accordance with the Declaration of Helsinki [23] and with the Swiss laws (Human Research Act and Human Research Ordinance). This study was approved by the medical ethics research committee Basel and registered with the Ethikkomission Nordwest- und Zentralschweiz (EKNZ) [BASEC 2016-00002] and the German Clinical Trials Register [DRKS00010418]. All forms given to patients and information obtained using the above mentioned questionnaires have been approved by the EKNZ. Essential changes in the course of the trial will be reported immediately and submitted for approval by the ethics committee.

Information and results will not be presented to the EKNZ on a regular basis, but, for data verification, authorized representatives of the project manager and the ethics committee have access at any time to the medical data relevant to the project, including the medical history of participants.

Serious adverse events must be reported immediately, and if potential life-threatening complications occur, the trial will be stopped unless the safety is proven by the ethics committee. Patients participating in this clinical trial are covered by a special hospital insurance. This insurance is free for patients and covers any damage or potential damage as well as death caused by the study.

## Results

The trial was started in June 2016 and enrolled 50 patients by article publication. Regarding the adherence to our protocol, we found 10% of loss of follow-up until now. Only 2 patients needed reoperation and only 1 patient needed a change of treatment (antiseptic therapy). Most patients are happy with their randomized treatment but as expected some patients in the sterile group complained about timing problems with their working hours and home-care service appointments. Most patients in the nonsterile group are satisfied being able to take care of their wounds independently although some patients still depend on the home-care service for the wound dressing.

We are hoping to have enrolled enough patients by summer 2017. The follow-up will take until autumn 2017, and study results are expected to be published by the end of 2017. This trial is solely supported by the cantonal hospital of Lucerne.

## Discussion

To date, the standard in postoperative aftercare for surgically drained soft tissue abscesses remains under debate. As patients are often young, it is important that they can return and participate as soon as possible in daily (working) life after an operation.

A more independent postoperative treatment plan could lead to an increase in independence during the healing process and lead, based on a more self-perceiving view of the patient, to better results in terms of quality of life during the healing process. Moreover, a more independent wound treatment could lead to more cost-effective medicine, a more reasonable use of medical infrastructure, and an earlier return to work. This prospective randomized trial is therefore important in order to investigate whether a recommendation for nonsterile and independent wound irrigation is justifiable or not for surgically drained soft tissue abscesses. Moreover, if a nonsterile after-treatment protocol is justifiable, it is expected that it would improve quality of life and lead to a significant medical cost reduction of 300,000 CHF annually in our hospital.

## Abbreviations

ED: emergency department
EKNZ: Ethikkomission Nordwest- und Zentralschweiz
EQ-5D: EuroQol 5-Dimension Questionnaire
POSAS: Patient and Observer Scar Assessment Scale
SF-36: Short Form Health Survey
VAS: Visual Analog Scale
VSS: Vancouver Scar Scale
